# Editorial

**DOI:** 10.1002/nop2.62

**Published:** 2016-08-21

**Authors:** Roger Watson

## 1 Why bother reviewing?

I wrote this on my way to the 2016 meeting of the International Association of Nursing Editors (INANE) in London and the first session I attended was a meeting of Wiley nursing editors. One of my colleagues from Wiley had asked me if I would be prepared to speak and to be videoed for 5 min on the topic of reviewing. Specifically, she asked me if I would address the questions: ‘why do I review?’; and ‘how has being a reviewer helped my research career?’

I rarely turn down an opportunity to be videoed. I think that reviewing is important and, in this issue, Dr Parveen Ali from the University of Sheffield and I consider the peer review system and the variations that exist (Ali & Watson, 2016). However, I had not previously considered it in quite the way I was asked above. I used this editorial as an opportunity to collect my thoughts.

## 2 Why do I review?

My main motivation in reviewing manuscripts is curiosity, which means there is something in it for me. While I review most things that are sent to me, I ‘draw the line’ at predatory journals and I ensure that the area under research or the methodology in the manuscript really match my area of expertise. My first reaction on receiving a manuscript to review is to wonder what the person has done, what new information the manuscript contains and how well do they understand the methods they have applied. On that final point, I sometimes find that they understand the methods a great deal better than I; in these cases, I learn something. So, reviewing is helpful and it has helped me to develop what I do as well as advise others on what they do.

I do think we have a duty to review manuscripts; after all, others do it for us and if we did not undertake reviews then the process of peer review would stop and the publication of new information would slow down. I also value the review process. It is far from perfect but, compared with the alternatives – and there are some (Ali & Watson, 2016) – it is probably the best system we have. I know that, as well as spotting some excellent manuscripts and giving them my full support, I have occasionally spotted gross methodological errors, which would have been bad for the author and the journal had they got into print. Of course, I can only assume that the authors agree with my judgment and either re‐analyse the data or decide not to publish. One weakness of the peer review process and, indeed, of the academic publishing process is that we have no control over the subsequent submission to other journals and publication of manuscripts, we either reject or recommend for revision.

## 3 How has being a reviewer helped my research career?

Being a reviewer has helped me to write and publish better manuscripts. As much as I have pointed out methodical errors to other authors, I have had them pointed out in my own work. This has saved me from embarrassment and helped me to develop and, in one notable example, has led to collaboration with the reviewers who were kind enough to contact me outside of the reviewing process. Otherwise, my early involvement in reviewing and publishing led to my first invitation to serve on an editorial board and this was the start of my editing career. Between, starting this editorial and its completion, I was asked to review another manuscript. I hope I can approach this with the same enthusiasm and curiosity that I did my first review over 20 years ago.
